# The effects of sampling and instrument orientation on LiDAR data from crop plots

**DOI:** 10.3389/fpls.2023.1087239

**Published:** 2023-03-14

**Authors:** Azar Khorsandi, Karen Tanino, Scott D. Noble

**Affiliations:** ^1^ Department of Chemical and Biological Engineering, College of Engineering, University of Saskatchewan, Saskatoon, SK, Canada; ^2^ Department of Plant Sciences, College of Agriculture and Bioresources, University of Saskatchewan, Saskatoon, SK, Canada; ^3^ Department of Mechanical Engineering, College of Engineering, University of Saskatchewan, Saskatoon, SK, Canada

**Keywords:** wheat, LiDAR, phenomics, phenotyping, spatial sampling

## Abstract

Wheat is one of the most widely consumed grains in the world and improving its yield, especially under severe climate conditions, is of great importance to world food security. Phenotyping methods can evaluate plants according to their different traits, such as yield and growth characteristics. Assessing the vertical stand structure of plants can provide valuable information about plant productivity and processes, mainly if this trait can be tracked throughout the plant’s growth. Light Detection And Ranging (LiDAR) is a method capable of gathering three-dimensional data from wheat field trials and is potentially suitable for providing non-destructive, high-throughput estimations of the vertical stand structure of plants. The current study considers LiDAR and focuses on investigating the effects of sub-sampling plot data and data collection parameters on the canopy vertical profile (CVP). The CVP is a normalized, ground-referenced histogram of LiDAR point cloud data representing a plot or other spatial domain. The effects of sub-sampling of plot data, the angular field of view (FOV) of the LiDAR and LiDAR scan line orientation on the CVP were investigated. Analysis of spatial sub-sampling effects on CVP showed that at least 144000 random points (600 scan lines) or an area equivalent to three plants along the row were adequate to characterize the overall CVP of the aggregate plot. A comparison of CVPs obtained from LiDAR data for different FOV showed that CVPs varied with the angular range of the LiDAR data, with narrow ranges having a larger proportion of returns in the upper canopy and a lower proportion of returns in the lower part of the canopy. These findings will be necessary to establish minimum plot and sample sizes and compare data from studies where scan direction or field of view differ. These advancements will aid in making comparisons and inform best practices for using close-range LiDAR in phenotypic studies in crop breeding and physiology research.

## 1 Introduction

With an increasing global population, providing enough food to satisfy needs is a big challenge. Plant breeding has effectively increased agricultural productivity over the past century (Evenson and Golin, 2003). Connecting genotypes with their phenotypes and selecting high-yield and stress-tolerant plants can help crop breeders keep pace with population growth (Rahaman et al., 2015).

High-quality phenotypic data are vital to plant breeders’ decision-making process to realize genetic improvements (Chapman et al., 2014; Bai et al., 2016). Phenotyping methods are able to evaluate plants according to their different traits, such as physiology, yield, development, and tolerance to environmental stresses (Li et al., 2014; Rahaman et al., 2015). Some morphological traits that are often used to evaluate plant growth and characterize the canopy structure include canopy biomass (Hansen and Schjoerring, 2003; Ehlert et al., 2009), height (Zhang and Grift, 2012; Bendig, 2015), and leaf area index (LAI) (Baret et al., 2010; Béland et al., 2011; Béland et al., 2014; Verger et al., 2014; Zhao et al., 2015). Studies have shown that these morphological traits have a strong relationship with plant genotype, cultivars, growth rate and yield (Sharma and Ritchie, 2015; Friedli et al., 2016; Sun et al., 2018).

Biomass measurement is a good indicator of crop growth and growth rate, leaf area, organ size and partitioning and morphological characteristics. These data can be used to calculate radiation use efficiency and metabolite analysis (Pask et al., 2012). Biomass production can be reduced dramatically by stresses, resulting in a reduced ability of the crop to intercept solar radiation and a decrease in the photosynthesis rate and/or radiation use efficiency. Identifying genotypes that are able to maintain their biomass production during stress conditions is an essential key to finding the better-adapted lines (Pask et al., 2012).

Plant height has been used as a proxy for plant biomass (Madec et al., 2017) and can be a trait for phenotyping. Studies showed stress conditions affect the stem height that defines plant height (Ota et al., 2015; Tilly et al., 2015). Some individual traits, such as plant height and stem solidness, both have a beneficial relationship with plant yield and harvest index. (Pask et al., 2012). Blonquist et al. (2009) used height as one of the model’s inputs to evaluate the water stress condition in plants. These traits are good for breeders to screen large plant populations (Pask et al., 2012).

Traditional methods to measure such phenotypic traits are focused on single time points and therefore do not consider the developmental dynamics of these traits. The limited sampling possible for human evaluators is insufficient to capture the variation within plots (Guan et al., 2018). Using modern technologies to develop high-throughput phenotyping methods is a way to overcome traditional manual methods’ temporal and sampling limitations.

One technology that can provide 3D canopy data for estimating plant traits is LiDAR. LiDAR uses the phase shift between an emitted signal and the reflected return signal (or signals) to estimate the distance between the instrument (zero point) and a target. While the application of LiDAR for estimation of height and above-ground biomass has been well-established in forestry (Lucas et al., 2008; Eitel et al., 2013; Kankare et al., 2013; Greaves et al., 2015), the use of this technology in field crops is much less mature. Recent studies established the LiDAR scanning approach to estimate the number of spikes and crop density (Saeys et al., 2009). In most studies, LiDAR data are often presented in the form of a meshed, 3D reconstruction of the scanned surface. They have focused on extracting and estimating a variety of canopy information such as height, canopy biomass, leaf area, leaf shape, leaf inclination angle, leaf area index (LAI) and leaf area density (LAD) from these data (Ehlert et al., 2010; Gebbers et al., 2011; Tilly et al., 2014; Jimenez-Berni et al., 2018; Qiu et al., 2019; Su et al., 2019; Walter et al., 2019; Maesano et al., 2020).

However, some work has represented plot data using relatively simple histograms, representing the canopy vertical profile (CVP) (Jimenez-Berni et al., 2018; Furbank et al., 2019). To determine the histogram of the vertical height of points with respect to the ground, the distance between the ground and the LiDAR sensor must first be determined. One approach is to consider the peak of the histogram (i.e., mode) as the ground elevation for a given plot, assuming that some ground is visible through the canopy or at the plot edge (Jimenez-Berni et al., 2018). Evaluating the CVP of crop plots is a promising approach for providing information about plant processes and development, especially if these traits can be tracked throughout the growing season. In this study, LiDAR data from a canopy were height corrected, combined across an area of interest (plot), and presented as a CVP plot of height versus a normalized number of returns. One of the main questions here is how CVP or LiDAR histogram data can be affected by instrumental adjustments and data acquisition approach.

The main objective of this study was to evaluate factors that could impact the consistency of LiDAR data for creating repeatable CVPs for wheat. The particular objectives were completed as described below.

1) Find the minimum sample size to consistently capture the CVP characteristics of a wheat genotype per plot.
2) Ascertain the effect of the angular FOV of LiDAR on the resulting CVP, and
3) Determine the impact of scan line orientation with respect to the row direction.

## 2 Methodology

This study was conducted in two parts. Part one was a field experiment (2019) to investigate the spatial sampling and FOV effects on LiDAR data. Part two was a container experiment (2020) to investigate the repeatability and effect of direction of travel on LiDAR data.

### 2.1 Area of study

This study was carried out in Saskatoon, Saskatchewan. Data were collected in separate experiments in 2019 and 2020. The 2019 experiment included four wheat varieties (Stettler, Superb, AC Sadash and Acadia) planted on May 24, 2019, in well-watered and drought treatment blocks with three replicate plots of each variety (24 plots total). Due to rainfall patterns, these treatments were not substantially different. The plots were 2.5 m long and 1 m wide, and each plot contained five rows of wheat plants with a row spacing of 0.2 m.

The 2020 experiment consisted of a single replicate of two wheat varieties (Stettler and Superb) planted in three rows on July 26, 2020, under two irrigation treatments (well-watered and drought). These varieties were planted at the same time in 45-litre containers 56 cm long × 41 cm wide × 33 cm deep.

### 2.2 Data acquisition

**Figure 1:**
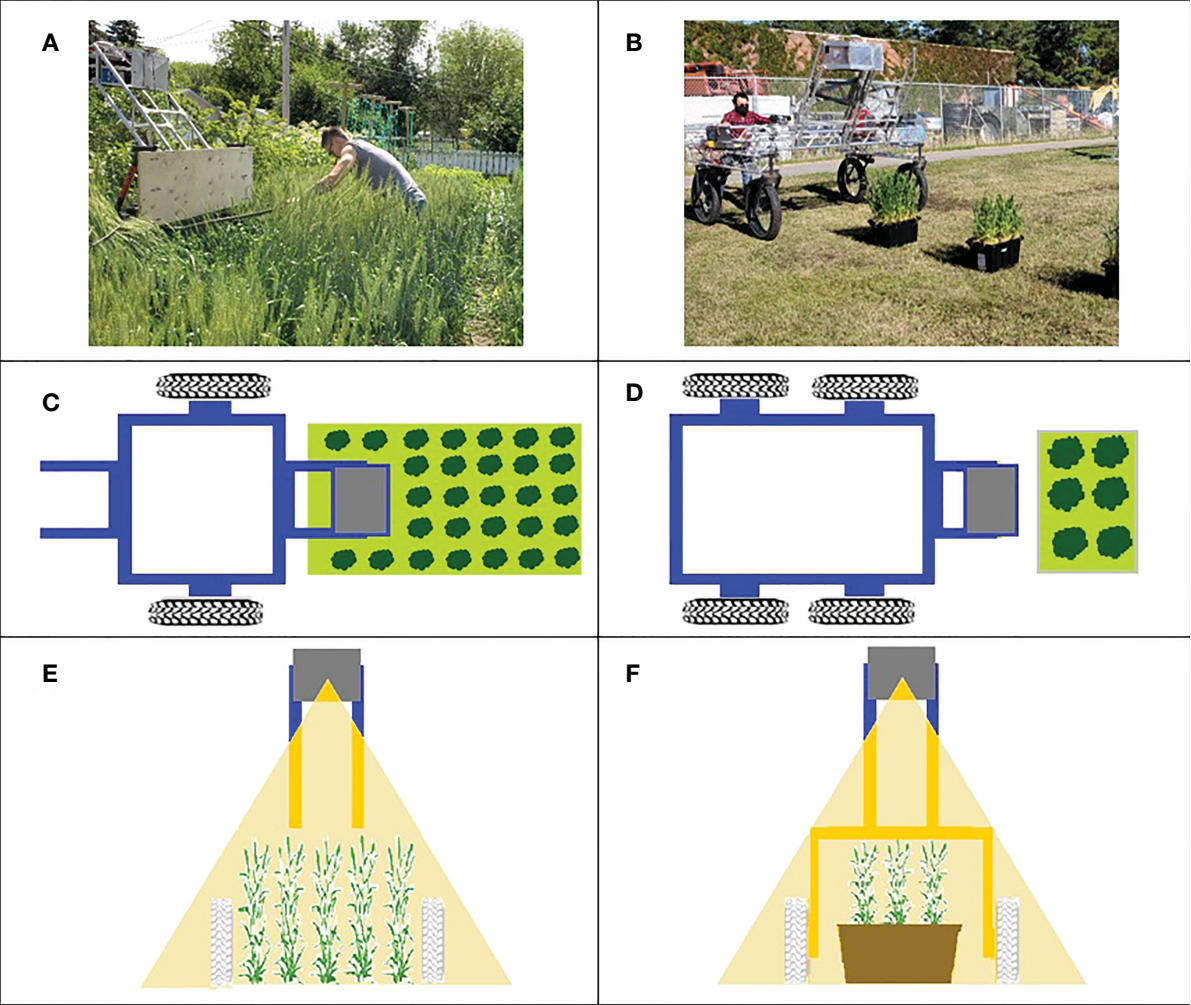
Data acquisition platform. Left column **(A, C, E)** shows the system used in 2019 experiment, right column **(B, D, F)** shows the 2020 system. Top row: photographs of the systems in use. Middle row: a top view diagram of data acquisition for the year. Bottom row: Front view showing the plane of the LiDAR scan.

In the 2019 experiment, the LiDAR scanner was mounted in the instrument payload on a two-wheeled cart consisting of a lightweight extruded aluminium frame that was pushed by an operator, manually (**Figures 1A, C, E**). In this experiment, the distance between the LiDAR scanner and the ground was inconsistent during data acquisition due to instabilities in the two-wheeled cart used. The 2019 experimental plots were organized in field layouts of columns and rows. Each data acquisition experiment included six scanning passes in the planting direction. Data were acquired in a single direction of travel. In this experiment, LiDAR scanning was conducted on 53 and 83 days after planting (DAP) when wheat varieties were at anthesis -Zadoks growth scale 61 [ZGS 61 (Zadoks et al., 1974)]- and ripening -ZGS 91-, respectively.

In the 2020 experiment, the instrument payload was mounted on the University of Saskatchewan Field Phenotyping System (UFPS) which is a portable cart consisting of a lightweight extruded aluminium frame with four wheels that was better able to maintain a consistent distance above the plants (**Figures 1B, D, F**). In this experiment, UFPS was pushed manually by the operator. Three data acquisition passes (replicates) were conducted for each canopy orientation (rows parallel and rows perpendicular to the direction of travel) on September 9, 10 and 11, 2020 (45, 46 and 47 DAP, respectively) when wheat varieties were at anthesis (ZGS 61). In both the 2019 and 2020 experiments, the operator pushed the cart.

The LiDAR sensor used (model SICK LMS 400-1000, SICK AG, Waldkrich, Germany) was a line scanning type. In this study, the FOV of the LiDAR was adjusted to 60°, and the scanning rate was 360 Hz with an angular resolution of 0.1°. The LiDAR working range was from 0.7 to 3 meters. The average speed of the cart carrying the LiDAR sensor was 0.23 m/s, resulting in an average interval between each scan line of 0.6 mm. The UFPS PhenoDAQ software was used for data acquisition.

### 2.3 Pre-processing

Raw data from the LiDAR were stored in HDF5 format on the payload computer. LiDAR scans contain information on range and return intensity (remittance) with 240 data points per scan line. The number of scan lines for each plot varied with the speed of travel and small variances in the lengths of plots.

The processing code was written in MATLAB (MATLAB R2018a). In the first step, raw LiDAR data were transformed from polar coordinates into Cartesian coordinates. In this step, each point comprises X, Y and Z coordinates, where X is the position along the direction of travel, Y is the position across the plot width (scan line orientation), and Z is the vertical position of each point.

The LiDAR data were collected in the LiDAR frame of reference; part of pre-processing is to convert data to a ground frame of reference. When the variation in the distance between the instrument and the ground was slight, the ground elevation for a given plot was considered the histogram mode (Jimenez-Berni et al., 2018). Subtracting the mode from the histogram data (and multiplying by -1), the histogram is transformed into a ground, rather than instrument, frame of reference. This step was called height correction pre-processing, and the resulting graph was the histogram of distances from the ground. To make this histogram comparable between different sample size, normalization with the number of return points were conducted, and the resulting graph was named CVP.

**Figure 2:**
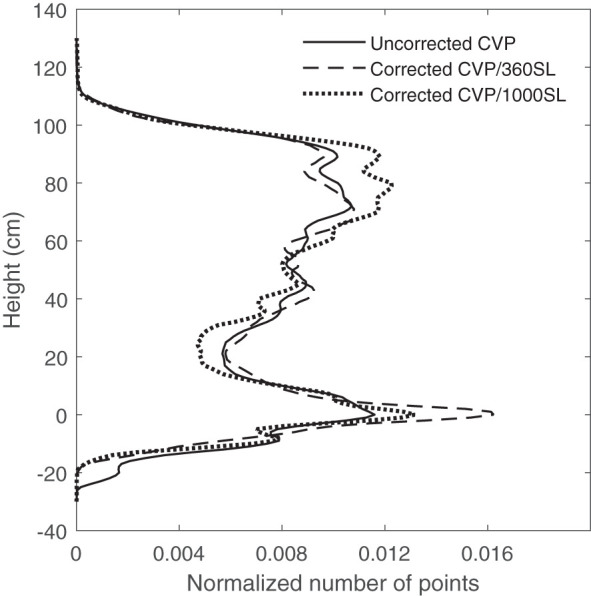
The effect of the different number of contiguous scan lines for applying height correction pre-processing on ground peak elevation on CVP.

When the distance between the ground and the instrument is inconsistent, the peak at the ground distance is less distinct when data are observed in aggregate for a plot. This issue was corrected by applying the height correction pre-processing to small numbers of contiguous scan lines at a time and aggregating them post-correction. This process was called ground correction pre-processing. In this pre-processing, it was assumed that variation of payload height with respect to the ground was negligible over a time of 1 second. The data collection frequency in this study was 360 Hz which means 360 lines were scanned with the LiDAR sensor within each second. **Figure 2** shows the effect of the different numbers of contiguous scan lines for applying height correction pre-processing on appearing ground peak elevation on CVP data compared with uncorrected CVP data. As can be seen in **Figure 2**, the raw height corrected aggregate CVP data ground peak is broad, having been spread out by variations in instrument height. Applying the height correction pre-processing on every 360 contiguous scan lines and then aggregating these post-correction resulted in a more distinct ground peak. Applying the same process on a larger number of contiguous lines did not produce the sharp ground peak seen in the ground correction using 360-scan line blocks. As it takes several seconds to collect 1000 scan lines, there may be more variation in instrument height over that more prolonged time, resulting in a less defined peak. Negative height values in these CVP graphs were created due to the instrument uncertainty and the variation in the surface profile and instrument height (**Figure 2**).

### 2.4 Impacts of LiDAR spatial sampling

The number of LiDAR data points is influenced by the size of the plot, LiDAR scan rate, angular resolution, and travel speed. A practical question for high-throughput phenotyping with LiDAR is how large a sample (both in terms of data points and area) is required to consistently capture the CVP characteristics of a larger plot. These sampling questions were studied using LiDAR data from the 2019 field data.

In the 2019 experiment, each treatment was planted in triplicate with a plot size of 2.5 m^2^. Plant growth in these plots was observed to be relatively uniform. Thus, the data were combined for each variety yielding four aggregate plots per treatment. Each aggregate plot was equivalent to 7.5 m^2^ containing roughly 375 wheat plants in five rows with approximately 12,000×240 LiDAR points. The CVP obtained from this aggregate plot was considered the reference CVP for the variety. Each wheat plant in these aggregate plots occupied an average of 0.02 m^2^ of space. These data were subsampled in two ways: random points and contiguous blocks.

To find the minimum number of randomly sampled points required to accurately estimate the CVP of an entire plot, random subsamples were taken, and CVP’s compared to the CVP from the entire plot. Subsamples were taken using numbers of points ranging from 24×10^3^ (equal to 100 scan lines) to 24×10^5^ (equal to 10000 scan lines) and CVPs were constructed. This was repeated three times for each number of points. The resulting CVPs were normalized with the number of points, and then their standard deviations across the canopy height were investigated. In the next step, each CVP was compared to that made by using the entire population in terms of the root mean squared error (RMSE).

For the spatially contiguous subsets, blocks of scan lines were selected from the total population based on the nominal in-row space required for between one and 12 plants along the rows. Like the random selection case, the resulting CVPs were visually compared to the aggregate plot CVP and used the root mean squared error (RMSE).

### 2.5 Field of view in LiDAR and its effect on CVP

**Figure 3:**
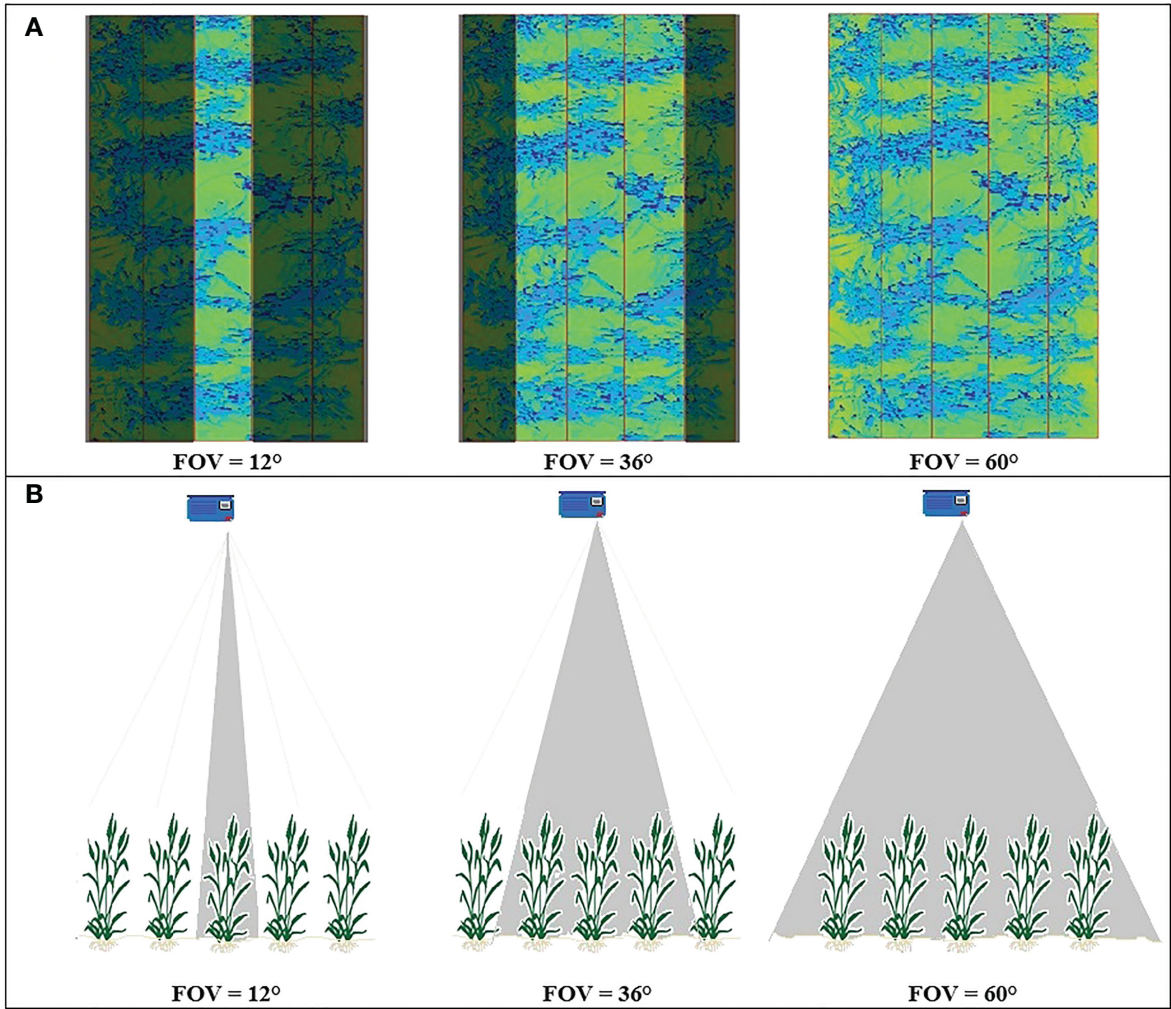
**(A)** the LiDAR images obtained from a typical plot on 15/08/2019, highlighting the scanned region covered by different angular FOV. **(B)** a representation of the FOV geometry viewed perpendicularly from the scanning plane.

It was hypothesized that the angular FOV of the LiDAR influences the resulting CVP of a scanned canopy. In the 2019 experiment, each plot contained five rows of wheat scanned with the LiDAR FOV of 60° (**Figure 3**). These data were used to create three FOV scenarios: 12°, 36°, and the entire 60° FOV. These roughly equated to scanning only the middle row of the plot, the centre three rows, and the whole plot, respectively (**Figure 3**). The resulting data were processed for each of the 24 plots, and then compared.

### 2.6 Repeatability and Orientation

To investigate the repeatability of the CVP of the scanned canopy, an experiment was conducted with four containers of wheat planted in rows. Two containers were planted with “Superb” and the other two with “Stettler”. One container of each variety was designated as a well-watered treatment and the other as a water-stressed (deficiency) treatment. Plants in the well-watered group were regularly watered in the days prior to measurements, while the water-stressed group was allowed to dry out. The containers were placed in a line with their rows aligned and scanned with the LiDAR scanner. Then each container was turned 90°, so their rows were perpendicular to the cart’s travel direction, and they were scanned again. Rotating the containers 90° and scanning them was repeated six times. In total, three passes of LiDAR measurements were made for the rows aligned with the cart’s travel direction and three passes were made for the rows perpendicular to the cart’s travel direction. This experiment was repeated on September 9, 10 and 11, 2020, with both treatments being thoroughly watered following scanning on September 9. The CVP of each container and measurement replication was obtained from the LiDAR data and compared to observe the repeatability of LiDAR canopy measurements and the effect of row orientation on the CVP data.

## 3 Results and discussion

### 3.1 Pre-processing

**Figure 4:**
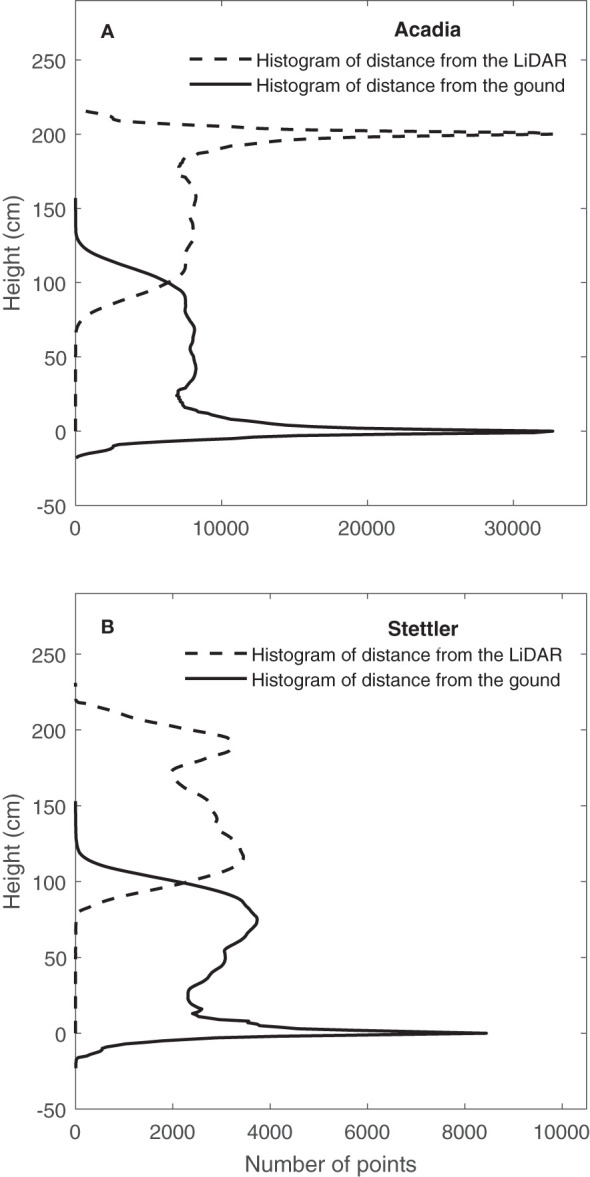
Determination of ground elevation from LiDAR data on 15/08/2019. Histogram of distance from the LiDAR and CVP after distance correction and height correction pre-processing were applied for a typical plot **(A)** (Acadia) when the distance between LiDAR and ground was consistent and **(B)** (Stettler) when there was variation in payload height with respect to the ground.

After converting the LiDAR data from polar coordinates to Cartesian coordinates, the histogram of distance from the LiDAR sensor was provided (**Figure 4**). One typical result on 15/08/2019 for one Acadia wheat plot (**Figure 4A**) showed that, in this plot where payload height with respect to the ground was consistent, the ground elevation peak could be clearly seen in the histogram of distance from the LiDAR (Jimenez-Berni et al., 2018). **Figure 4A** shows that in this typical case, the ground correction process did not affect the shape of the histogram, but height correction pre-processing corrected the height of points in the histogram of distance from the ground.

In contrast, results on the same date but for a typical Stettler wheat plot lacked a distinct ground peak, indicating an inconsistent distance between the LiDAR and the ground (**Figure 4B**). In this case, the ground correction process’s effect can be observed in **Figure 4B**. In general, results showed that after this pre-processing, the ground elevation peak was significant and sharp on the histogram of distance from the ground (**Figure 4B**). Overall, after doing ground correction pre-processing, height correction pre-processing was conducted, and CVP for the plots was determined.

### 3.2 Impacts of LiDAR spatial sampling

**Figure 5:**
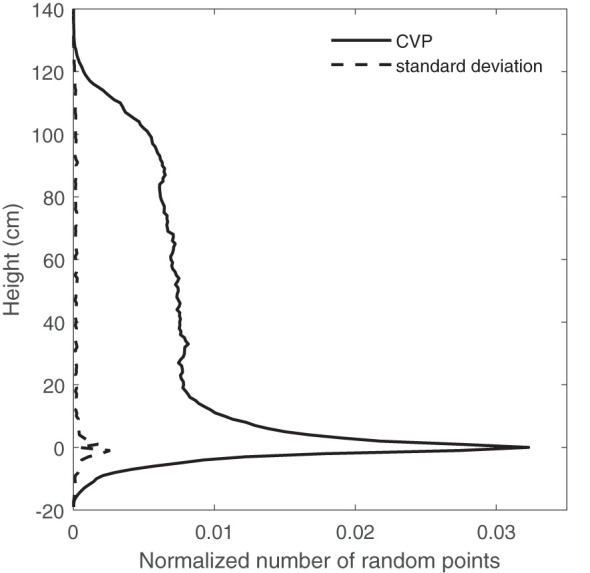
The average of 10 CVPs obtained from 800×240 random points and the standard deviation of these CPV graphs- in different canopy heights.

A comparison of CVP graphs obtained from different numbers of random points showed a lot of variation around the ground peak area. **Figure 5** is one case that shows the standard variation of the normalized number of points for an average of 10 CVPs obtained from 192000-point random samples (equal to 800 scanlines). The most considerable standard deviations in the CVP graphs were related to the ground peak, and its neighborhood (-10 to +10 cm) shows the variations of obtained CVPs around the ground peak are much larger than those across the canopy height. This might happen due to the uneven ground surface or the existence of dead leaves or litter on the ground surface. Including or excluding data from the ground peak region on the overall RMSE when comparing a subsampled CVP to a reference may be critical to evaluating subsampling performance. This was assessed as part of the determination of minimum subsample size.

**Figure 6:**
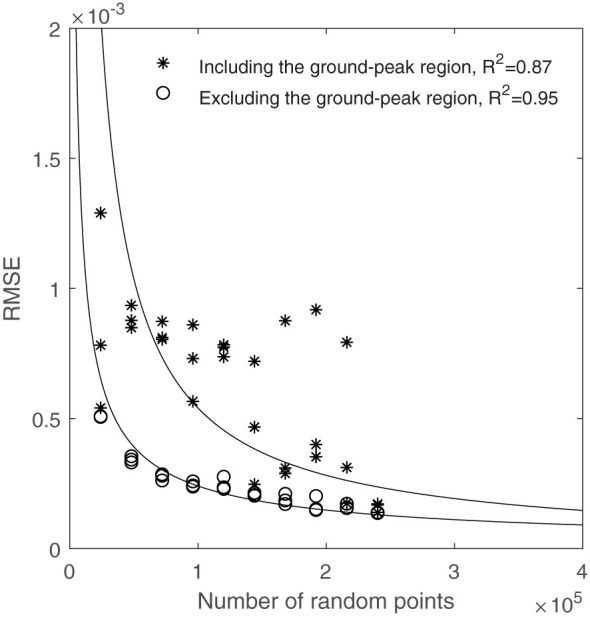
The relationship between the number of random points used and RMSE compared to using the whole area points, including and excluding the ground-peak region.

**Figure 7:**
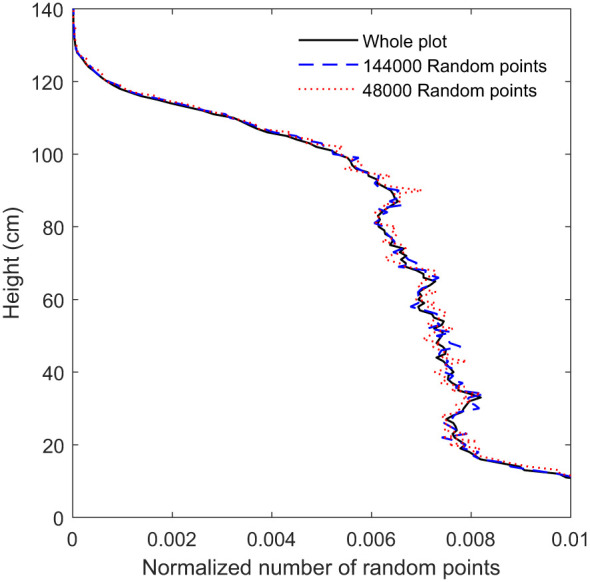
The CVP obtained from LiDAR data provided with 48000, 144000 and whole plot data.

**Figure 6** shows the calculated RMSEs between each CVP graph and the reference CVP made using the entire population. With the majority of subsample variance occurring in the region of the ground peak, this analysis was conducted both including and excluding the ground-peak region ( ± 10cm). A stronger relationship between RMSE and the number of random subsample points was found for CVP’s with the ground peak excluded (R^2 =^ 0.95) than with these data included (R^2 =^ 0.87). In addition, **Figure 6** shows that most outlier points disappeared by removing the ground peak neighborhood points from the RMSE calculation process. **Figure 6** illustrates the RMSE decreased with a power relationship with an increasing number of random points in the subsample. The incremental improvement in CVP representation with increasing subsample size decreases rapidly. Comparisons of selected subsample CVPs with the whole-plot reference are shown in **Figure 7**.

As seen in **Figure 6**, RMSE related to the 144000 random points (equal to 600 scan lines) is near the shoulder in the curve, and below this amount of random points, RMSE increased rapidly. **Figure 7** shows that the CVP obtained from 144000 random points was very similar to that obtained from the whole plot area. In contrast, the CVP obtained from 48000 random points (equal to 200 scan lines) had lots of variation and could not follow the reference CVP. For this reason, 144000 random points were selected as the minimum random subsample points to capture the CVP characteristics of a larger plot.

**Figure 8:**
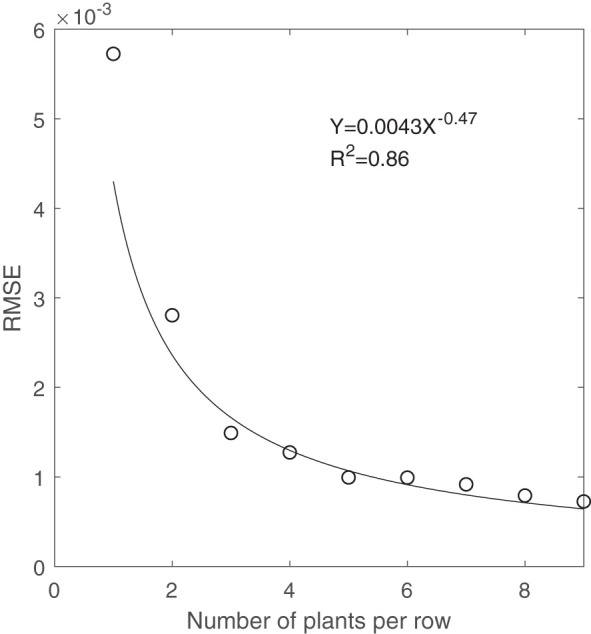
The correlation between areas containing the different number of plants per row and RMSE.

**Figure 9:**
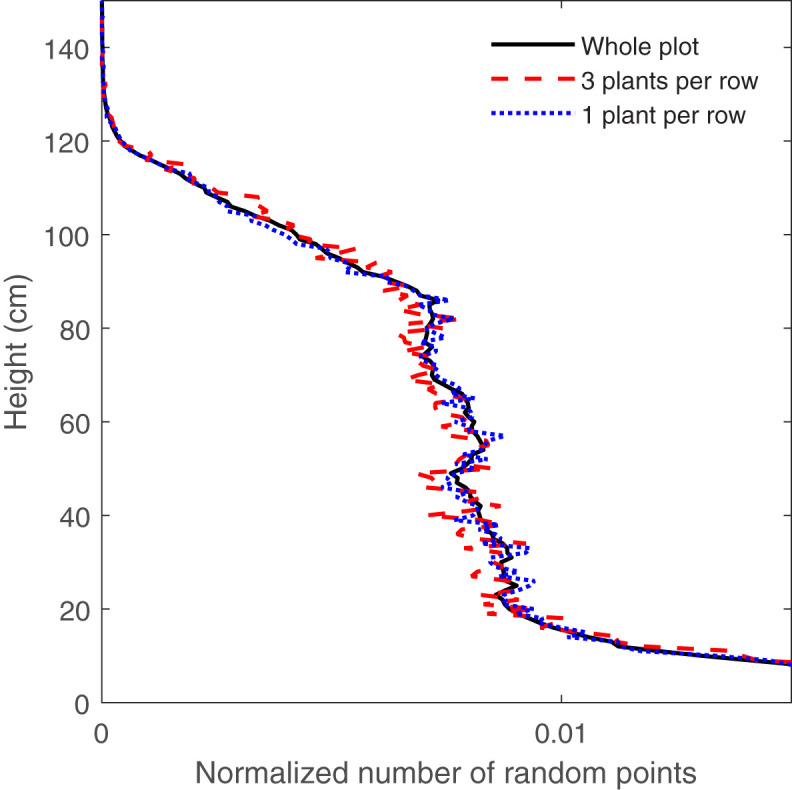
The CVP obtained from LiDAR data provided with three different numbers of plants per row.

Similarly, **Figure 8** shows that with increasing the number of plants per row scanned by the LiDAR scanner, RMSE also decreased with a power relationship. Reducing the number of plants below three, RMSE changed dramatically from 0.0015 to 0.003, suggesting three plants as the minimum sample extent for predicting changes in the CVP of the whole population scanned by LiDAR. In addition, **Figure 9** shows that the CVP of scanned data with three plants per row could follow the shape of the CVP provided by the whole plot data. The average number of normalized points on each height for the area with the entire population, three-plant length section and one plant length section was 0.006. It was concluded that the area containing three or more plants per row was adequate to characterize the overall CVP of the aggregate plots.

### 3.3 The effect of LiDAR field of view on CVP

**Figure 10:**
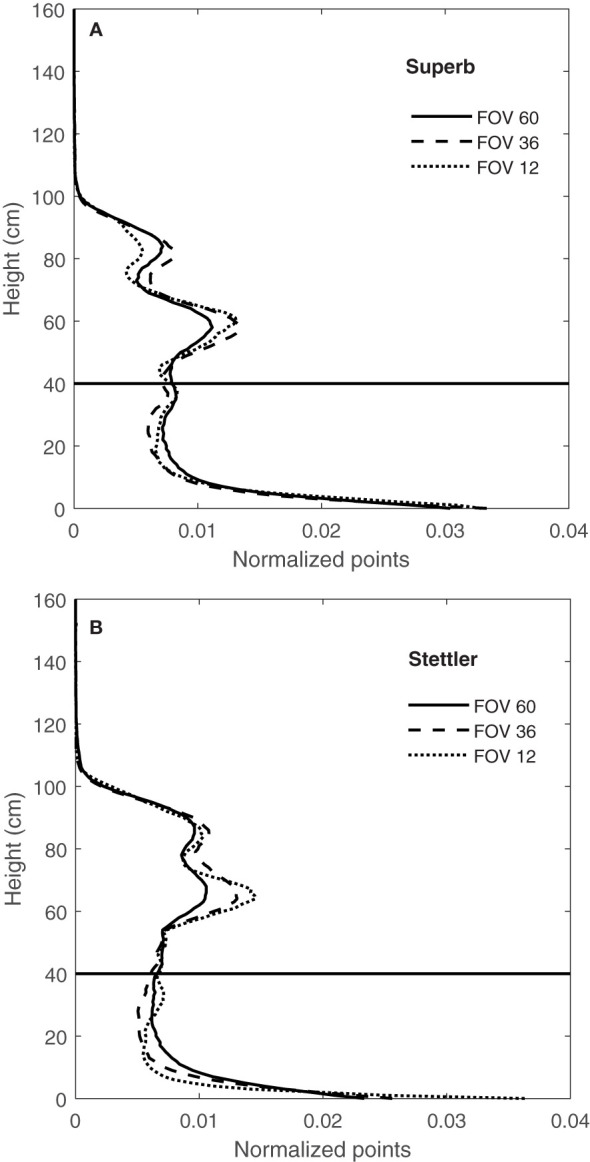
CVPs for FOV of 12, 36 and 60° for wheat genotype Superb **(A)** and for the wheat genotype of Stettler **(B)** on 15/08/2019 and the difference in the proportion of areas for FOV 12 and 60° for height greater than 40 cm.

A comparison of the CVPs for three FOVs of 12, 36 and 60° showed changes in the shapes of CVPs with FOV (**Figure 10**). It was observed that responses varied with the angular range of the LiDAR data, with narrow ranges having a more significant proportion of returns in the upper canopy and a lower proportion of returns in the lower part of the canopy.

This difference in normalized CVPs with the number of points at different FOV might be due to the direction of LiDAR’s rays hitting the canopy. As shown in **Figure 3**, at the narrow 12° FOV, the LiDAR was more-or-less directly over the middle row of the plot, so the middle 12° of the scan primarily sees the top of the row. In this case, the upper parts of the canopy block the path of the rays and prevent them from penetrating the lower parts of the canopy. Therefore, the proportion of points related to the upper levels of the canopy is greater at this FOV and lower in the bottom half of the canopy (**Figures 10A, B**). With a wider 60° FOV, the inter-row space helps make a gap in the canopy allowing off-nadir rays to penetrate deeper into the canopy (**Figure 3**). This results in more information being collected from the sides of the plants compared to a narrower FOV. This increases the proportion of the CVP area lower in the canopy (**Figures 10A, B**). The 36° FOV acted more like a narrow FOV, and the proportion of points related to the upper levels of the canopy was greater at this FOV and lower in the bottom half of the canopy (**Figures 10A, B**).

### 3.4 Repeatability results

**Figure 11:**
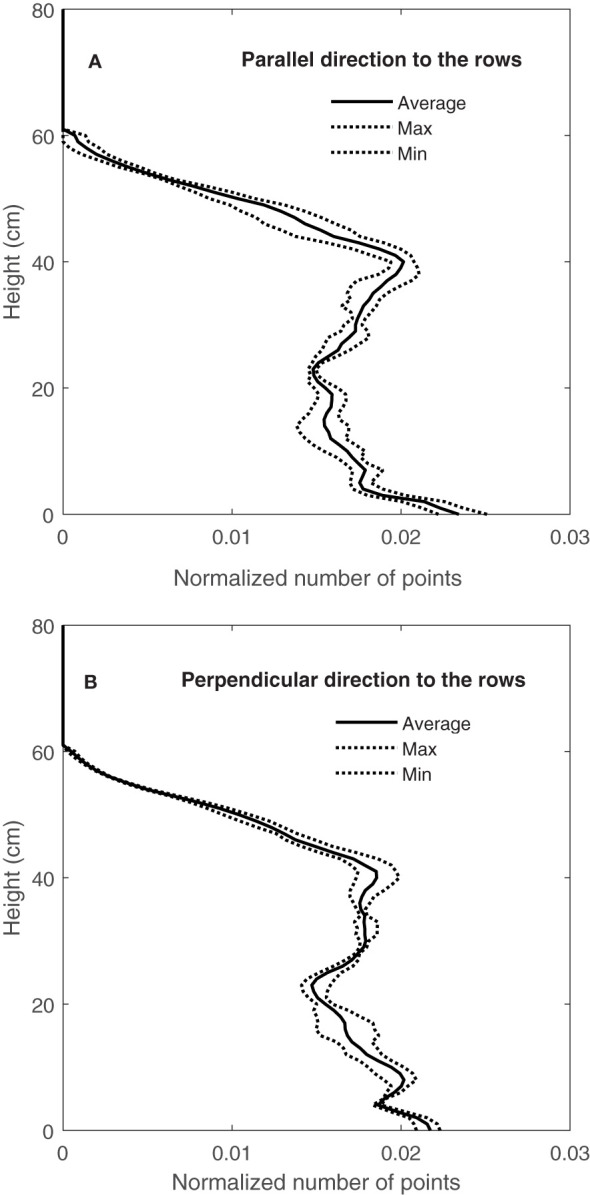
Variation of normalized CVP for two directions of travel **(A)** parallel to (or with) the rows and **(B)** perpendicular to (across) the rows.

**Figure 11** shows the variation in CVPs obtained by LiDAR from well-watered Superb wheat grown in a container and scanned with the direction of travel parallel to the rows (**Figure 11A**) and direction of travel perpendicular to the rows (**Figure 11B**). Both directions of travel (parallel to and perpendicular to the rows) showed a similar amount of CVP variation. Similar results were observed in CVPs obtained from other containers on September 9, 10 and 11, 2020.

**Figure 12:**
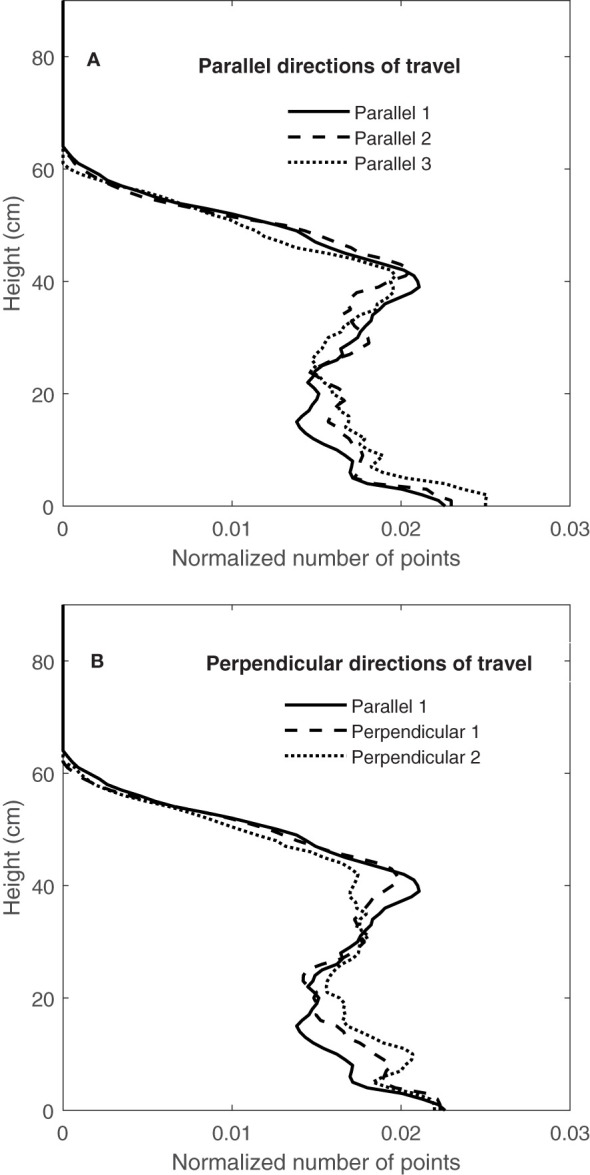
Comparing the CVP obtained from **(A)** the same directions of travel and **(B)** the perpendicular directions of travel from one container on 09/09/2020.

**Figure 12A** shows the comparison of three scans, obtained from travelling parallel to the rows (parallel 1, 2 and 3), and **Figure 12B** shows the comparison of orthogonal scans, one with a travel direction parallel to the rows (parallel 1) and the other two with travel direction perpendicular to the rows (perpendicular 1 and 2). Results showed that CVPs obtained from parallel directions of travel (**Figure 12A**) and perpendicular directions of travel, were followed each other in peaks and height. Similar results were observed in other containers on consecutive days (September 9, 10 and 11). These results showed the repeatability of LiDAR data in two perpendicular directions of travel for the same containers.

**Figure 13:**
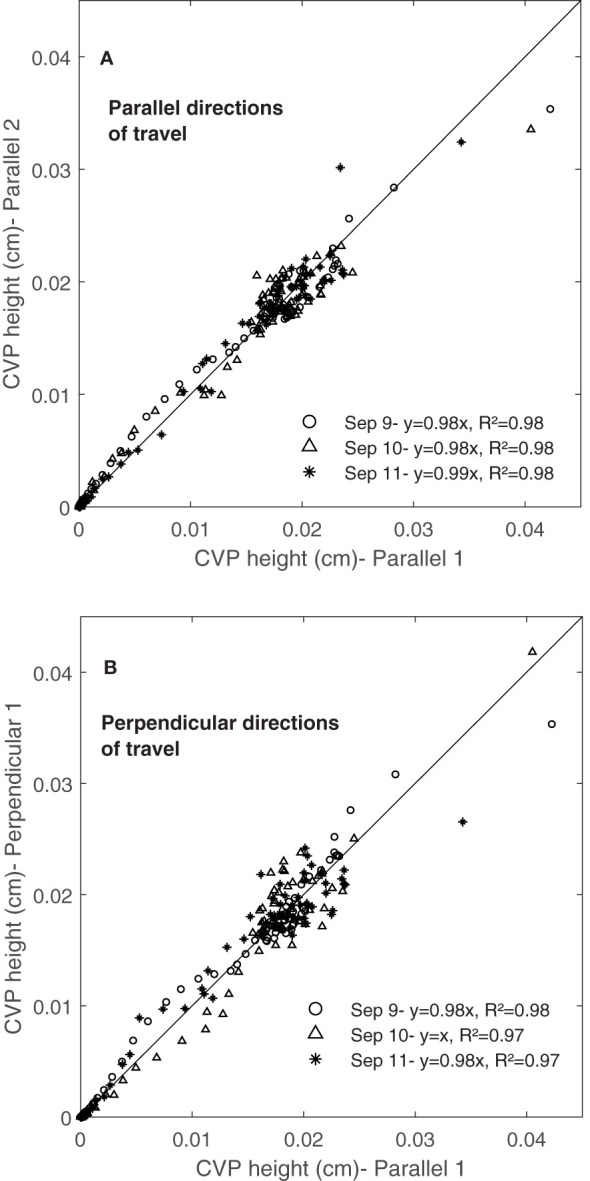
The Repeatability of CVP graphs obtained in three consequence days. Comparing scans collected **(A)** from the same directions of travel and **(B)** from the perpendicular directions of travel during three days (9, 10 and 11 of September 2020) from the same container.

**Figures 13A, B** show the repeatability of CVPs on consecutive days (September 9, 10 and 11) again for the well-watered Superb container by plotting CVP pairs against each other. Comparing the same travel direction (with respect to the rows), the linear regression slope coefficients were near one, and the intercepts were near zero (**Figure 13A**). However, comparing orthogonal travel directions (parallel 1 and perpendicular 1) in **Figure 13B**, scanned data showed much greater variation with a lower linear regression coefficient than the same directions. Similar results were observed for the other three containers on September 9, 10 and 11. Walter et al. (2019) conducted an experiment to investigate the repeatability of LiDAR measurements. Their results showed that the repeatability of LiDAR measurements was higher in the same directions of travel than in opposite directions of travel.

## 4 Conclusion

In this study a ground-based LiDAR system was used to collect data from wheat plots, from which histograms of height vs normalized number of points were constructed and referred to as the canopy vertical profile (CVP). Height correction pre-processing and normalization with a number of return points were two main steps to convert histogram data to CVP and make them ready and comparable for the subsequent analysis. However, when the distance between the ground and the instrument was inconsistent, applying ground correction pre-processing to small numbers of contiguous lines (360) at a time and aggregating them post-correction was a solution to convert the LiDAR data from an unsteady sensor to the ground frame of reference.

This study showed that the CVP of a scanned, uniform plot could be represented by a subset of at least 144000 random points (600 scan lines). In addition, analysis of the impact of LiDAR spatial sampling showed that areas containing at least three plants per row are needed to consistently capture the CVP characteristics of wheat genotype per plot.

Investigating the impact of LiDAR FOV \ on CVP graphs showed differences between narrow and wide fields of view. The narrow 12° FOV of the scan rays primarily sees the top of the canopy in a row directly below, preferentially returning top-of-canopy points. In wider FOVs, the off-nadir rays can penetrate deeper into the canopy profile and provide more information from the lower parts of the canopy due to the inter-row space and the gaps that happened in the canopy. This observation confirms that LiDAR FOV influenced the CVP graph and should be considered during data acquisition and comparing results from different instruments or scan settings.

Multiple measurements of CVP of the same canopy were shown to be repeatable when collected from the parallel or perpendicular travel directions with respect to the rows. These advancements may help plant breeders to compare data from studies where scan direction, FOV, or sample sizes differ. This combination of findings demonstrates the ability of LiDAR to provide repeatable information about the vertical profile of wheat plants in field conditions. In future studies, the ability of CVP as a phenotypic trait can be investigated by comparing the relationship between the CVPs obtained from different plant genotypes with other plant traits.

## Data availability statement

The original contributions presented in the study are included in the article/supplementary material. Further inquiries can be directed to the corresponding author.

## Author contributions

AK: author, design of the work, analysis, interpretation of data, and drafting of the work. KT: co-author and funding. SDN: author, design of the work, interpretation of data, and revising. All authors contributed to the article and approved the submitted version.
