# Corrigendum: The effects of sampling and instrument orientation on LiDAR data from crop plots

**DOI:** 10.3389/fpls.2023.1217158

**Published:** 2023-05-29

**Authors:** Azar Khorsandi, Karen Tanino, Scott D. Noble

**Affiliations:** ^1^ Department of Chemical and Biological Engineering, College of Engineering, University of Saskatchewan, Saskatoon, SK, Canada; ^2^ Department of Plant Sciences, College of Agriculture and Bioresources, University of Saskatchewan, Saskatoon, SK, Canada; ^3^ Department of Mechanical Engineering, College of Engineering, University of Saskatchewan, Saskatoon, SK, Canada

**Keywords:** wheat, LiDAR, phenomics, phenotyping, spatial sampling

In the published article, there was an error in the Funding statement. This research was also funded by the “Western Grains Research Foundation” and should be added to the list of funders. The previous Funding statement was “The authors acknowledge the financial support of the Dean’s Scholarship and from the College of Graduate and Postdoctoral Studies (CGPS), University of Saskatchewan., the Saskatchewan Ministry of Agriculture, Saskatchewan Wheat Development Commission, and the Canada First Research Excellence Fund *via* the P2IRC project.”. The correct Funding statement appears below.

